# Anti-Inflammatory Activity of Bioaccessible Fraction from *Eryngium foetidum* Leaves

**DOI:** 10.1155/2013/958567

**Published:** 2013-09-17

**Authors:** Suwitcha Dawilai, Chawanphat Muangnoi, Phawachaya Praengamthanachoti, Siriporn Tuntipopipat

**Affiliations:** Institute of Nutrition, Mahidol University, Salaya, Nakhon Pathom 73170, Thailand

## Abstract

*Eryngium foetidum* (EF) has long been used as a medicinal plant and culinary spice in tropical regions. Phytochemicals in its leaves have been proposed to be responsible for the anti-inflammatory and antioxidant activities. The present study used *in vitro* digestion coupled with Caco-2 cells to assess such activities. Caco-2 cells were incubated with aqueous fraction from simulated digestion (bioaccessible fraction) of EF leaves with/without bile extract prior to stimulation with interleukin-1 beta (IL-1*β*). Monocyte chemoattractant protein-1 (MCP-1) and IL-8 in culture media and the intracellular reactive oxygen species (ROS) were measured. Approximately 24% *β*-carotene and 35% lutein of leaves were present in the aqueous fraction. The transfer of caffeic and chlorogenic acids to the aqueous fraction was 76%–81%, while that of kaempferol was 48%. Prior incubation of Caco-2 cells with the bioaccessible fraction suppressed IL-1*β* activated IL-8 and MCP-1 by 33%, but the fraction lacking mixed micelles decreased IL-8 and MCP-1 levels only by 11%. The pretreatment of Caco-2 cells with the bioaccessible fraction of EF reduced ROS by 34%; the fraction lacking mixed micelles decreased ROS by 28%. These data suggest that bioactive compounds partitioning in mixed micelles play a significant role to suppress the proinflammatory insult but with a modest antioxidant effect.

## 1. Introduction

Intestinal epithelial cells engage in various activities such as the absorption of dietary nutrients and their metabolites and providing physical and biological barriers to gut microbiota, antigens, and xenobiotics. In response to stimuli such as invasion by pathogenic bacteria, hydrogen peroxide, and inflammatory cytokines during inflammatory response, intestinal epithelial cells also secrete inflammatory cytokines and chemokines [1, 2]. Excessive production of such mediators during chronic inflammatory conditions disturbs the gut homeostasis causing onset of intestinal disorders including inflammatory bowel diseases (IBDs) [3–5]. IL-8 or CXCL8, an *α*-chemokine, is highly expressed in the intestinal mucosa in IBD [6], facilitating persistent infiltration by neutrophils [7]. The expression level of IL-8 was correlated with the disease activity [8]. Monocyte chemoattractant protein-1 (MCP-1) or CCL2 is another chemokine that recruits monocytes, memory T cells, and dendritic cells to the affected tissue during inflammation [9, 10]. Elevation of MCP-1 expression is also observed in the mucosa of IBD patients [9, 11] and contributes to the pathogenesis of various immunodeficiency and inflammatory diseases [12]. Reactive oxygen species (ROS)/reactive nitrogen species (RNS) and biomarkers of oxidative injury are elevated, and intestinal mucosal antioxidants are reduced in IBD patients compared to control subjects. The extent to which the antioxidant levels and oxidative stress biomarkers are altered has been associated with the severity of intestinal inflammation in IBD patients [13]. Although blocking the activity of cytokines represents one therapeutic strategy for IBD [14, 15], the cost [16] and undesirable side effects [17] limit this approach. Natural compounds in diets continue to be of interest for use as an alternative approach to treat IBD due to their relative safety reason and low cost. The immunomodulatory and anti-inflammatory properties of bioactive compounds, and in particular polyphenolic compounds derived from dietary agents including fruits and vegetables, have been investigated [18, 19].

*Eryngium foetidum *L. (EF) which is also referred to as Mexican coriander or fit weed, is cultivated in the West Indies and tropical regions including Thailand. It has been traditionally used to treat fevers, colds, and flu [20]. Its leaves are used as a flavor in many recipes due to its pungent aroma [21, 22]. Based on the food consumption of Thailand, the mean consumption at 97.5 percentile of the Thai population is 18.75 g/person/day in 19–35 years old of eater only population [23]. The leaves contain 0.1–0.95% dry weight essential oil, predominantly consisting of *E*-2-dodecenal [24]. Beside the essential oil, triterpenoids, carbonyls, alcohols, and terpenes have been identified in *E. foetidum* leaves [25]. A crude extract from EF leaves was reported to have anti-inflammatory activity in murine macrophage cell lines [26]. A hexane extract of the leaves enriched with stigmasterol also was reported to exhibit topical anti-inflammatory activity against acute and chronic inflammation induced by topical application of 12-*O*-tetradecanoylphorbol acetate (TPA) on a mouse's ear [27]. Ingestion of a decoction of *E. foetidum* leaves also showed inhibition of both carrageenan-induced paw edema and TPA-induced ear edema in rodents [28]. Although *E. foetidum* leaves have been previously reported to exhibit topical anti-inflammatory activity, anti-inflammatory activity as an actual consumption has not been reported. The objectives of this study were to determine the anti-inflammatory activity and the ROS scavenging capacity of digested EF leaves. Leaves were subjected to simulated gastric and small intestinal digestion, and the aqueous or bioaccessible fraction was separated from the undigested material in chyme. The bioaccessible fraction was added to cultures of Caco-2 human intestinal cells activated with IL-1*β* to simulate an inflammatory insult. This cell model is widely used to investigate the transport, metabolism, and efficacy of dietary compounds in foods and metabolites generated during digestion [29]. We also investigated the impact of the deletion of the bile extract during the small intestinal phase of digestion to determine the contribution of fat soluble compounds in the digested leaves on anti-inflammatory properties in the Caco-2 cell cultures.

## 2. Materials and Methods

### 2.1. Chemicals and Reagents

Dulbecco's modified Eagle's medium (DMEM), pepsin, porcine bile extract, porcine pancreatin, porcine lipase, and protease from a bovine pancreas were purchased from Sigma Chemical Co. (St. Louis, MO, USA). L-glutamine, nonessential amino acids, penicillin-streptomycin, and Fungizone were obtained from Invitrogen (Grand Island, NY, USA). Fetal bovine serum (FBS) was purchased from PAA Laboratories (GmbH, Pasching, Austria). All chemicals were either analytical grade or high performance liquid chromatography (HPLC) grade. All chemicals were obtained from Sigma Chemical Co. Human IL-8, MCP-1 capture, biotin-labeled detection antibodies, and human IL-1*β* were purchased from Peprotech Inc (Rocky Hill, NJ, USA).

### 2.2. Sample Preparation

*E. foetidum* leaves were purchased from major distributors in Bangkok, Chiang Mai, Ubon Ratchathani, and Nakhon Pathom province. Fresh *E. foetidum* leaves (EF) were washed with tap water and rinsed with deionized water. The root was simply cut off to obtain the edible portion (approximately 77% of the whole leaf). The edible portions were chopped into 2 inches length and snapped frozen with liquid nitrogen prior to vacuum packing and maintaining at −20°C. The sample was further frozen at −80°C overnight prior to lyophilization. Dried samples were homogenized with an electric blender with equal quantities of prepared materials from the four distributors pooled prior to storage in aluminum foil in vacuo at −20°C.

### 2.3. *In Vitro* Digestion

Simulated gastric and small intestinal phases of digestion were performed according to Garrett et al. [30] and Ferruzzi et al. [31]. Digestion reactions contained 0.7 g freeze dried with 3% (v : wt.) soybean oil. After completion of simulated digestion, the digested samples were centrifuged (Becton Dickinson Dynac Centrifuge, Sparks, MD, USA) at 10,000 g for 1 h at room temperature to isolate the aqueous fraction. Bile extract was omitted in some reactions to determine whether the lipophilic compounds in the extract that require incorporation into mixed micelles during the small intestinal phase of digestion contributed to the activity of the bioaccessible fraction. Control digestions without EF extract were also performed to assess the possible effects of the compounds in the bioaccessible fraction on cellular activities. The supernatant was collected and filtered through a 0.22 *μ*m polytetrafluoroethylene (PTFE) membrane (Millipore Corporation, Cork, Ireland). The sample headspace was blanketed with nitrogen gas prior to storing at −80°C until HPLC analysis within one week. Filtered aqueous fractions were analyzed as below and added to differentiated cultures of Caco-2 cells to assess bioactivity and uptake of carotenoids and phenolic content in Caco-2 cells.

### 2.4. Extraction and Analysis of Carotenoids

Carotenoids in freeze dried sample, digesta, and the aqueous fraction were extracted according to Olives Barba et al. [32]. Briefly, samples were extracted with hexane : acetone : ethanol (50/25/25, v/v/v), sonicated in an ultrasonic bath at 67 kHz and 85 Watt (Mettler Electronics Corp., Anaheim, California, USA) for 10 min and centrifuged at 5,000 g for 10 min. The supernatant was evaporated with a rotary evaporator (Buchi Rotavapor-Re-124, Flawil, Switzerland) at 38–40°C. The dried extract was resolubilized in 1–3 mL of mobile phase solution and filtered (0.22 *μ*m PTFE membrane). The extract was further diluted to an appropriate concentration with the mobile phase prior to analysis by HPLC (Agilent 1100 series, Santa Clara, CA, USA). The HPLC protocol for carotenoid analysis has been described elsewhere [33]. The concentration of each carotenoid was quantified compared with a standard containing lutein, zeaxanthin, lycopene, *α*-carotene, and *β*-carotene.

### 2.5. Extraction and Analysis of Phenolic Compounds

One gram of freeze dried EF leaves was extracted with 10 mL of 6 M HCl and 40 mL of 62.5% methanol containing 0.5 g/L tert-butylhydroquinone (TBHQ) [33] in a shaking water bath at 70°C for 2 h. The samples solution was placed in a cold water bath for 5 min, added with 100 *μ*L of 1% (w/v) ascorbic acid solution and adjusted volume to 50 mL with methanol in a volumetric flask. The extract was sonicated in an ultrasonic bath for 5 min and filtered with 0.2 *μ*m PTFE membrane (Millipore Corp., Cork, Ireland) prior to analysis by HPLC. The digesta and aqueous fraction were extracted with equal volume of absolute ethanol twice, sonicated for 10 min, and centrifuged at 5,000 g for 10 min. The combined supernatants were evaporated with rotary evaporator at 38–40°C until dryness. Dried extract was hydrolysed with acid methanol (6 mL deionized water : 4 mL 6 M HCl) and 10 mL methanol containing 0.5 g/L tert-butylhydroquinone (TBHQ) and incubated in a shaking water bath at 70°C for 2 h. Samples were placed on ice for 5 min, added with 1% (w/v) ascorbic acid solution, and brought up to 20 mL with absolute methanol. The extract was sonicated for 5 min and filtered through 0.22 *μ*m PTFE membrane into an amber vial prior to analysis by HPLC. High performance liquid chromatograph (Agilent Technologies 1100 series coupled with a photodiode array detector) was equipped with a Zorbax Eclipse XDB-C18 column (4.6 × 150 mm, inner diameter 5 mm, Agilent Technologies) and preceded by a cartridge guard column with the same packing. The mobile phase consisted of 100% water containing 0.5% (w/w) trifluoroacetic acid (solvent A), 100% methanol containing 0.5% (w/w) trifluoroacetic acid (solvent B), and 100% acetonitrile containing 0.5% (w/w) trifluoroacetic acid (solvent C). The column temperature was controlled at 30°C. The phenolic content was separated by the gradient elution programs as previously described [34] and identified by comparison of retention time and spectra with pure standards (chlorogenic acid, caffeic acid, ferulic acid, taxifolin, vitexin, naringin, naringenin, myricetin, morin hydrate, quercetin, luteolin, hesperitin, kaempferol, apigenin, and isorhamnetin). The content was quantified at 338 nm by comparing peak areas with calibration curves.

### 2.6. Cytotoxicity Test

Caco-2 cells were purchased from the American Type Culture Collection (ATCC, Rockville, MD, USA) and used between passages 24 and 35. Cells were seeded and maintained in complete medium as previously described [30]. The cytotoxicity of bioaccessible fraction with EF on IL-1*β* activated Caco-2 cells was determined in a pilot experiment in order to select nontoxic amounts to assess functional properties. Monolayers of differentiated Caco-2 cells were incubated with diluted filtered aqueous fractions generated during digestions for 4 h prior to exposing cells to 10 ng/mL recombinant human IL-1*β* for an additional 20 h. Viability of treated cells was assessed by sulforhodamine B (SRB) assay [35]. Caco-2 cells were washed with phosphate buffered saline (PBS) before initiating the SRB assay by measuring absorbance at 500 nm. The absorbance was proportional to cell number and cultures with corrected absorbance >90% that of the control samples (e.g., no EF dried material added to the digestive reaction tube).

### 2.7. Anti-Inflammatory Activity of Bioaccessible Fraction on Inflamed Caco-2 Cells

Caco-2 cells were maintained 11–14 days after the monolayer attained confluency. To initiate an experiment, the monolayer was washed with a basal medium prior to addition of 2 mL of bioaccessible fraction diluted 1 : 3 with basal DMEM and incubated for 4 h prior to stimulation with IL-1*β* for another 20 h. Culture media were collected to measure IL-8 and MCP-1 by ELISA. In brief, high binding plates (NUNC, Roskilde, Denmark) were incubated with appropriate concentration of capture antibodies against IL-8 and MCP-1 overnight before incubating with 1% bovine serum albumin (BSA) in PBS for 2 h. After washing with PBS with 0.05% Tween 20 (PBST), various concentrations of recombinant IL-8 and MCP-1 standards and appropriate dilutions of culture medium were added to each well and incubated in a moist chamber at 4°C overnight. The plates were washed with PBST prior to incubation with biotinylated antibody against IL-8 and MCP-1 for 2 h. After washing, the antigen-antibody complex was captured with streptavidin horseradish peroxidase (HRP)-tetra methyl benzidine detection system (Pierce, Rockford, IL, USA). Sulfuric acid (1 N) was added to terminate reactions, and the absorbance at 450 nm was determined by a microtiter plate reader (TECAN, Grödig, Austria). The concentrations of IL-8 and MCP-1 were calculated by comparing absorbance with the curve generated with the standards.

### 2.8. Intracellular Reactive Oxygen Species (ROS) Measurement

After the 4 h pretreatment of cultures with diluted bioaccessible fraction, the monolayer was washed and stimulated with IL-1*β* for 20 h. The treated monolayer was washed with warm PBS prior to addition of 5 *μ*M dichlorofluorescin diacetate (DCF-DA) (Sigma) and incubated at 37°C for 30 min. Cells were washed 3 times with warm PBS and lysed with 0.5% Triton X-100 in cold PBS. The supernatant of lysate cells was obtained by centrifugation at 14,000 g for 5 min at 4°C. The fluorescent signal in the supernatant was measured using the excitation wavelength at 485 nm and emission wavelength at 530 nm by Luminescence Spectrometer LS55 (Perkin Elmer Instruments LCC, Shelton, CT, USA).

### 2.9. Data Analyses

SPSS version 13 was used for statistical analyses. All parameters were conducted in triplicate, and each experiment was performed at least 2 times. The descriptive statistics including the mean and the SD were calculated for percent bioaccessibility (proportion of carotenoids or phenolic compounds present in the filtered aqueous fraction relative to those contained in the food sample), IL-8, MCP-1, and ROS. Means were analyzed by ANOVA when appropriate followed by Tukey's multiple comparisons (values with *p* < 0.05 were considered significant).

## 3. Results

### 3.1. Quantity of Carotenoids and Phenolic Compounds in Aqueous Fraction

**Figure 1:**
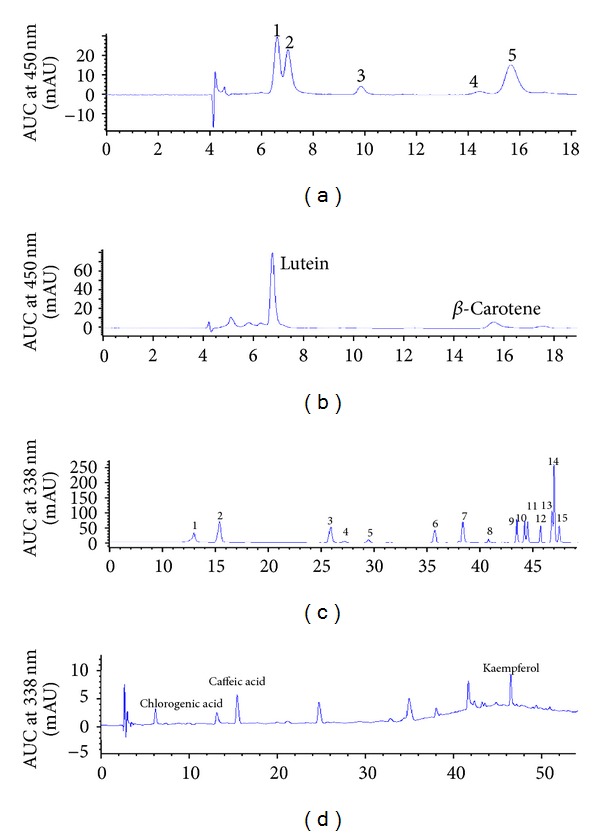
(a) Chromatogram of carotenoid standards: (1) lutein, (2) zeaxanthin, (3) lycopene, (4) *α*-carotene, and (5) *β*-carotene. (b) Chromatogram of carotenoids from *E. foetidum* leaves. (c) Chromatogram of phenolic compound standards: (1) chlorogenic acid, (2) caffeic acid, (3) ferulic acid, (4) taxifolin, (5) vitexin, (6) naringin, (7) naringenin, (8) myricetin, (9) morin hydrate, (10) quercetin, (11) luteolin, (12) hesperitin, (13) kaempferol, (14) apigenin, and (15) isorhamnetin. (d) Chromatogram of phenolic compounds from *E. foetidum* leaves.

**Figure 2:**
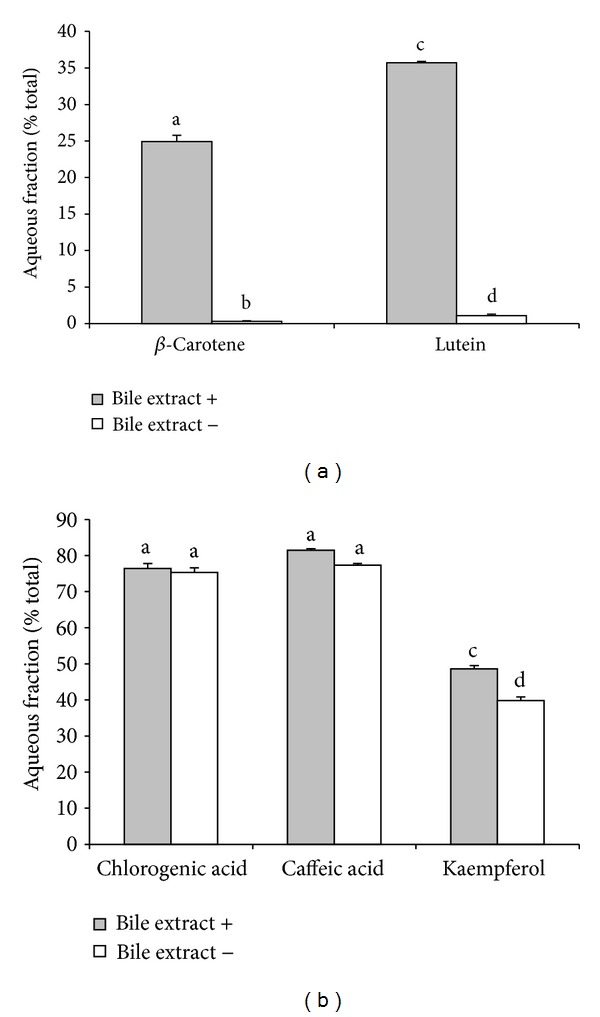
Efficiency of the transfer of BC and LUT (a) and CA, CGA, and kaempferol (b) from EF leaf to the filtered aqueous fraction during simulated digestion with or without bile extract. Efficiency represents the percentage of the amounts of BC, LUT, CA, CGA, and kaempferol in *E. foetidum* leaf transferred to the filtered aqueous fraction during simulated digestion. Data are mean ± SD; *n* = 6. Means without a common letter above the error bar differ significantly (*p* < 0.05).

*E. foetidum *leaf contained twice as much lutein (692 g/g dry wt) as *β*-carotene (BC: 326 *μ*g/g dry wt) (Figure 1(b)). *E. foetidum* leaf also contained several phenolic compounds including caffeic acid (CA: 209 *μ*g/g dry wt), chlorogenic acid (CGA) or 5-O-caffeoylquinic acid (338 *μ*g/g dry wt), and kaempferol (136 *μ*g/g dry wt) (Figure 1(d)). Additional peaks were also present in the chromatographic profile of *E. foetidum* leaf (Figures 1(b) and 1(d)), but the retention times and the absorption spectra did not match standards in the HPLC system (Figures 1(a) and 1(c)). Approximately 24.5% and 35.7% of BC and LUT in the lyophilized leaf were present in the filtered aqueous fraction. Deletion of the bile extract during small intestinal digestion significantly decreased BC and LUT content in the filtered aqueous fraction (Figure 2(a)). Approximately 81%, 76%, and 48% of CA, CGA, and kaempferol in the freeze dry leaf were present in the filtered aqueous fraction. In contrast to the carotenoids, deletion of bile extracts during small intestinal phase of digestion slightly, but significantly, decreased kaempferol in the filtered aqueous fraction without alter in the content of CA and CGA (Figure 2(b)).

### 3.2. Effect of Bioaccessible Fraction on IL-1*β* Induced Secretion of IL-8 and MCP-1 by Caco-2 Cells

**Figure 3:**
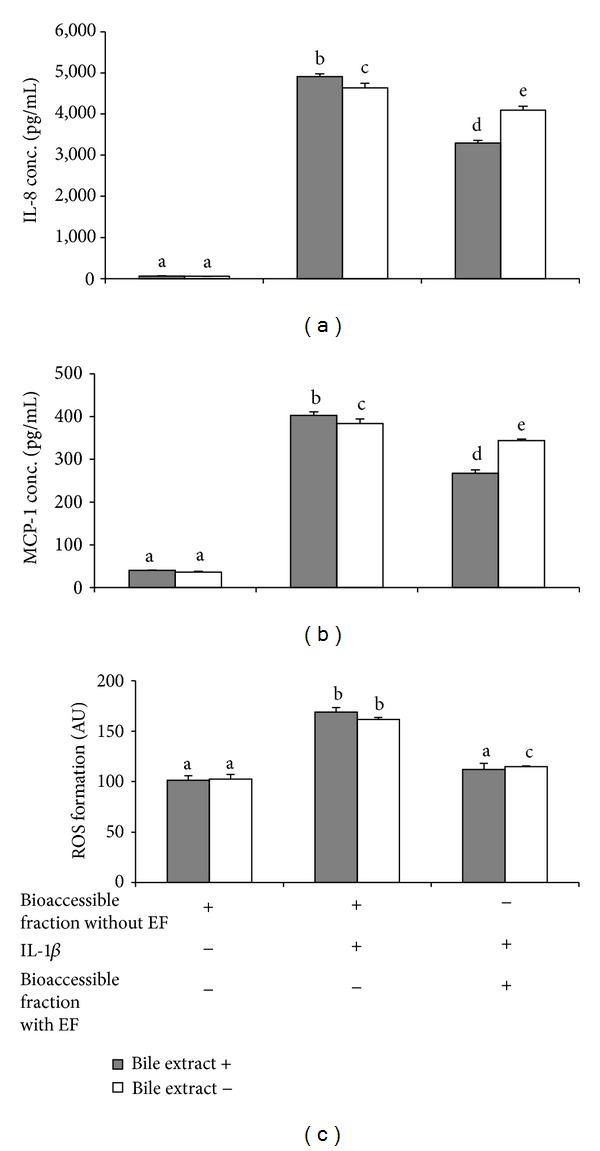
The effect of the bioaccessible fraction of EF generated in the presence and absence of bile extract during simulated digestion on secretion of IL-8 and MCP-1 and the intracellular ROS activity in IL-1*β* activated Caco-2 cells. In differentiated cultures, Caco-2 cells were incubated for 4h with diluted (1 : 3) bioaccessible fraction of digested EF generated with or without bile extract prior to the addition of IL-1*β*. Cell culture medium was collected after 20 h to quantify IL-8 (a) and MCP-1 (b). After washing with PBS, monolayers were incubated with DCF-DA before lysing with Triton X-100. Supernatant was collected to measure fluorescent as a marker for intracellular ROS as described in method (c). Results are mean ± SD for the three independent experiments. Means without a common letter above the error bar differ significantly (*p* < 0.05).

Cytotoxicity as assessed by SRB assay was not observed when Caco-2 cell monolayer was incubated with the bioaccessible fraction (i.e., the filtered aqueous fraction) generated during the simulated digestion of EF for 4 h prior to the addition of IL-1*β* to cultures for an additional 20 h (data not shown). Caco-2 cells exposed to 10 ng/mL IL-1*β* following the pretreatment with diluted bioaccessible fraction lacking EF secreted 80-fold more IL-8 into the medium than control cultures (4910 versus 61 pg/mL; *p* < 0.05). Preincubation with the diluted bioaccessible fraction from the digested EF decreased medium IL-8 in IL-1*β*-activated cells by 33% (*p* < 0.05) (Figure 3(a)). In contrast, deletion of bile extract during the small intestinal phase of digestion attenuated the anti-inflammatory activity of the bioaccessible fraction of digested EF (Figure 3(a)). Similarly, IL-1*β* activated Caco-2 cells secreted 10-fold more MCP-1 (402 pg/mL) than control cultures (40 pg/mL). Pretreatment of the Caco-2 cell monolayer with diluted bioaccessible fraction of EF for 4 h decreased MCP-1 secretion by 33% (*p* < 0.05) (Figure 3(b)). Deletion of the bile extract during digestion of EF leaves generated a bioaccessible fraction that only inhibited the secretion of MCP-1 by 10.7% (Figure 3(b)). These data suggest that the carotenoids and other lipophilic compounds from digested EF leaves that partition in mixed micelles are the primary factors responsible for the observed attenuation of the response of Caco-2 cells to IL-1*β*.

### 3.3. Bioaccessible Fraction of EF Decreases IL-1*β* Induced Intracellular ROS Accumulation

Pretreatment of Caco-2 cells with diluted bioaccessible fraction without digested EF significantly enhanced intracellular ROS in response to treatment with IL-1*β* (Figure 3(c)). When cultures were preincubated with the diluted bioaccessible fraction before exposure to IL-1*β*, intracellular ROS was decreased (*p* < 0.05) by 34% (Figure 3(c)). Similarly, incubation with diluted bioaccessible fraction of EF generated by digestion in absence of bile extract decreased intracellular IL-1*β* induced ROS by 28.5% (*p* < 0.05). These results indicated that the majority of the antioxidant activity in digested dried EF is due to hydrophilic compounds in the aqueous fraction and not carotenoids and other lipophilic compounds. 

## 4. Discussion

We previously demonstrated that ethanolic extract from EF leaves suppressed gene expression of IL-6, TNF-*α*, iNOS, and COX-2 and reduced intracellular ROS in LPS-activated murine macrophage cell lines [26]. The present study used the coupled *in vitro *digestion/Caco-2 cell model to determine whether the bioaccessible fraction of digested EF leaves retained anti-inflammatory and antioxidant activities. The results clearly show that the bioaccessible fraction of EF leaves decreased IL-1*β* induced secretion of IL-8 and MCP-1 as well as increased intracellular ROS production in Caco-2 cells. Although our data suggest that BC, LUT, CA, CGA, and kaempferol contributed to this anti-inflammatory activity, it is likely that there are other unidentified compounds in the bioaccessible fraction of EF also important for the attenuated response to the inflammatory insult. Due to lack of standards comparable with the unidentified peaks of chromatograms from *E. foetidum*'s leaf, we could not characterize such compounds. Our observation that the majority of the anti-inflammatory activity of the bioaccessible fraction of EF leaves was lost when the bile extract was deleted during small intestinal digestion which supports the importance of the delivery of lipophilic compounds in mixed micelle to the apical membrane of Caco-2 cells for attenuating the response to the inflammatory insult. 

Lutein was shown to inhibit H_2_O_2_ induced IL-8 expression in gastric epithelial AGS cell line [36]. Also, BC and LUT suppressed H_2_O_2_ induced increases in intracellular ROS, activation of NF-*κ*B, and IL-8 expression in gastric epithelial cells [36]. Feeding rats BC-rich alga supplemented diets for 10 weeks suppressed the basal and acetic acid induced mucosal myeloperoxidase activity and prevented acetic acid induced histopathologic changes in epithelial cells, edema in the lamina propria, infiltration of inflammatory cells, and hemorrhage [37]. Recently, carotenoids from dried pepper (*Capsicum annuum* L.) extract also showed antioxidant and anti-inflammatory effects [38]. Thus, anti-inflammatory activity in the intestinal cell model in the present study further supports the possible role of lutein and *β*-carotene in *E. foetidum* leaf in gut health.

Because the bioaccessible fraction generated in the absence of the bile extract retained a portion of the anti-inflammatory activity, CA, CGA, kaempferol, and other polar compounds in digested EF leaves also contributed to the suppression of IL-1*β* induced secretion of IL-8 and MCP-1. (Figures 3(a) and 3(b)). Both CA and CGA have been reported to downregulate TNF-*α* and H_2_O_2_ induced secretion of IL-8 by Caco-2 cells [39]. Chronically feeding mice CA for 10 weeks attenuated dextran sodium sulfate (DSS) colonic inflammation [40]. Kaempferol was also demonstrated to exert an anti-inflammatory activity as well [41–43]. C57BL/6J mice fed with 0.3% kaempferol had significantly reduced plasma NO, prostaglandin E2 (PGE2), and leukotriene-4 (LTB4) after treatment with DSS [43]. The same study reported that kaempferol downregulated the mucosal expression of TNF-*α*, IL-6, and IL-1*β* in DSS treated mice. 

The more polar bioactive compounds including CA, CGA, and kaempferol in the bioaccessible fraction of EF reduced intracellular accumulation of ROS in IL-1*β* treated Caco-2 cells. The presence of the lipophilic compounds in mixed micelles had minimal impact on the antioxidant activity. The antioxidant activity of CA and CGA has been demonstrated in intestinal ischemia-reperfusion injury in rats [44]. Antioxidant enzymes including glutathione peroxidase, superoxide dismutase, and catalase were increased in murine cardiac tissue after feeding diabetic mice a diet containing 2% CA [45]. CGA also was reported to markedly improve hepatic function and suppress oxidative stress by reducing ischemia/reperfusion induced liver injury, enhancing heme oxygenase-1, expression and activating nuclear factor erythroid-related factor 2 (Nrf-2) in rats [44]. Recently, rats fed with 200 mg kaempferol/kg body weight lowered the 1,2-dimethyl hydrazine induced erythrocyte lysate and liver thiobarbituric acid reactive substances level and recovered liver and colon antioxidant enzymes catalase, superoxide dismutase, and glutathione peroxidase [46].

## 5. Conclusions

Lipophilic compounds such as carotenoids that partition in mixed micelles generated during digestion of EF leaves had a major role in the anti-inflammatory activity in cultures of IL-1*β* treated Caco-2 cells. In contrast, phenolics and other polar compounds attenuated the oxidative stress induced by the inflammatory insult in Caco-2 cells. These data suggest that the consumption of EF fresh leaf may be a promising approach for preventing intestinal inflammation. A human intervention trial must be performed to determine if the suggestion is valid.
